# Perceptions of COVID-19 during and after the Omicron outbreak among healthcare personnel in Indonesia

**DOI:** 10.3389/fpubh.2023.1321045

**Published:** 2024-01-08

**Authors:** Mohammad Ainul Maruf, Yi-Hao Weng, Ya-Wen Chiu, Hung-Yi Chiou

**Affiliations:** ^1^Ph.D. Program in Global Health and Health Security, College of Public Health, Taipei Medical University, Taipei, Taiwan; ^2^Faculty of Public Health, Universitas Muhammadiyah Jakarta, Jakarta, Indonesia; ^3^Department of Pediatrics, Chang Gung Memorial Hospital, Chang Gung University College of Medicine, Taipei, Taiwan; ^4^Department of Public Health, College of Health Sciences, Kaohsiung Medical University, Kaohsiung, Taiwan; ^5^Research Center for Global Health and Security, Kaohsiung Medical University, Kaohsiung, Taiwan; ^6^Department of Medical Research, Kaohsiung Medical University Hospital, Kaohsiung, Taiwan; ^7^Institute of Population Health Sciences, National Health Research Institutes, Miaoli, Taiwan

**Keywords:** COVID-19, healthcare personnel, Indonesia, knowledge, attitudes, practices, psychological distress

## Abstract

**Introduction:**

The COVID-19 pandemic occurred in several waves with different levels of seriousness. Healthcare personnel (HCP) constituted a high-risk population for COVID-19, necessitating monitoring of their knowledge, attitudes, and practices (KAP) status and level of psychological distress. This study investigated differences in the impacts of COVID-19 during and after the Omicron outbreak among HCP in Indonesia.

**Methods:**

An online structured questionnaire survey was distributed twice in selected hospitals of Indonesia: the first survey was between December 2021 and February 2022 (Omicron era) and the second between August and October 2022 (post-Omicron era). A multiple logistic regression model was used to determine the differences in KAP and psychological distress among HCP toward COVID-19 with demographic characteristics adjusted for.

**Results:**

This study included 402 (Omicron era) and 584 (post-Omicron era) HCP members. Positive attitudes were more common in the Omicron era than in the post-Omicron era (*p* = 0.001). The availability of face shields and protective eyewear significantly decreased from 62.7 to 55.6% (*p* = 0.028). However, psychological distress among HCP significantly increased after the Omicron outbreak (*p* = 0.024). Multiple logistic regression analyses revealed a decrease of positive attitudes (OR = 0.626; 95% CI = 0.476–0.823) in the post-Omicron era.

**Conclusion:**

Our data indicated a significant increase in psychological distress among HCP in the post-Omicron era. These findings suggest a need for greater focus on psychological distress among HCP in Indonesia.

## 1 Introduction

The COVID-19 pandemic, which emerged in late 2019, has had a profound impact on societies worldwide, reshaping the way we live and work. The first case of COVID-19 in Indonesia was detected on March 2, 2020, in a patient who had been in contact with an individual with COVID-19 in Malaysia (1). Within a month, the virus had spread to 24 of the country’s 34 provinces and eventually caused several waves of outbreaks, including a second wave in mid-2021 by the highly contagious Delta variant (2, 3). Since August 2020, Indonesia had the highest number of COVID-19 cases in Southeast Asia and was ranked fourth in Asia (4). In December 2021, the Omicron variant was identified, further resulting in a significant increase in the number of cases. As of April 27, 2022, Indonesia has reported over 6 million cases and more than 150,000 deaths, making it the twenty-seventh country with the highest number of COVID-19 cases (5). Given the decline in the Omicron wave, Indonesia lifted its quarantine policy for foreign travelers and allowed mass gatherings since the end of March 2022 (Supplementary material S1). The COVID-19 pandemic has not only presented a substantial public health challenge, but has also ushered in unprecedented effects on the occupational environment. The global response to the pandemic, including measures such as lockdowns, social distancing, and the adoption of remote work arrangements, has fundamentally reshaped the dynamics of the workplace (6). The transition to remote work, propelled by the necessity for social distancing, has given rise to a range of psychological and physical challenges for workers across various industries (7).

In individuals of working age who have previously contracted COVID-19, some grapple with persistent symptoms termed post-acute COVID-19 sequalae or long COVID, which can significantly impact their productivity (8). This impact is not exclusive to general workforce but extends to healthcare personnel (HCP), thereby exerting strain on the healthcare system (9, 10). Positioned at the forefront of the battle against the virus, HCP encountered unique challenges and heightened risks (11, 12). Their perceptions toward COVID-19, including their knowledge, attitudes, and practices (KAP), play a crucial role in mitigating the spread of SARS-CoV-2 and ensuring the safety and mental well-being of both patients and HCP themselves (13). It is imperative to note that HCP, in particular, faced an elevated risk of virus exposure due to their direct contact with infected individuals.

In the course of performing their duties, HCP are required to use personal protective equipment (PPE). Prolonged use of PPE during the COVID-19 pandemic has resulted in discomfort and, more significantly, has led to serious dermatological problems among HCP (14). Excessive caution, prolonged working hours, and substantial workload have contributed to a high prevalence of stress, anxiety, and depression among HCP (15). A high prevalence of psychological distress, leading to burnout, may occur if the coping mechanisms undertaken by HCP are ineffective (16, 17).

Indonesia, a country with a high burden of COVID-19 cases and limited resources, has faced challenges in managing the pandemic, including among its HCP (11, 18). Understanding the perceptions toward the pandemic among HCP is critical to identifying areas for improvement, developing appropriate interventions, and preparing better strategies for the future outbreaks similar to COVID-19 in Indonesia and beyond. In addition, the variations in the severity of pandemic due to the ability of the virus to continue to mutate over time might cause changes in their KAP and pandemic prevention measures. Therefore, in the current study, we assessed the perceptions toward COVID-19 among HCP in Indonesia and identified the potential changes after the Omicron pandemic. Our study provides insights into the situation of COVID-19 pandemic on HCP in Indonesia, which can inform policies and interventions to support the healthcare system and protect the health of HCP.

## 2 Methods

### 2.1 Design

This study employed a repeated cross-sectional online survey design and was conducted first from December 2021 to February 2022 and then from August to December 2022. The first survey was conducted in the Omicron era: the Omicron variant was detected in December 2021 and its peak outbreak occurred in February 2022. The second survey was conducted when the number of COVID-19 cases in Indonesia declined after the Omicron outbreak. The questionnaire was structured and developed using questions from previous surveys (19–25), which were modified by five experts with backgrounds in medicine, statistics, and public health.

### 2.2 Participants

The questionnaires were targeted at HCP working in four hospitals in Indonesia (Cempaka Putih Jakarta Islamic Hospital, Syarif Hidayatullah Hospital, Malahayati Islamic Hospital, and Namira Islamic Hospital), which were selected using a cluster sampling method. Sample size calculation was not performed for this study, as the objective was to assess the perceptions of HCP in the selected hospitals, and the focus was on obtaining a representative sample from these specific institutions. The questionnaire was distributed as a Google Form survey, and data were collected accordingly. We contacted potential participants through each hospital’s Human Resources (HR) Department and the managers or heads of each unit, informed them about our study, and asked for their assistance in distributing the questionnaire link and/or QR code to their colleagues and subordinates. We also provided paper-based questionnaires in case potential respondents found it difficult to fill out the online questionnaires. The study excluded those who were not involved in health care and younger than 18 years old. The HR Department of Quality Control Department provided information on the total number of participants from each hospital.

### 2.3 Questionnaire

The survey contained questions on the participants’ demographic characteristics, KAP regarding COVID-19, institutional support, psychological distress, and experience of the impact of COVID-19 (Supplementary material S2). All items were rated on a 5-point scale, ranging from strongly agree to strongly disagree for questions on knowledge or attitudes and always to never for questions on practices, institutional support, psychological distress, and the pandemic’s impact. The demographic information collected included sex, age, profession (defined as medical job category), educational level, and whether participants held a faculty and/or a managerial position, lived with children or older adults, vaccination status, and experience in treating patients with confirmed or suspected COVID-19 and handling bodily specimens from patients with confirmed or suspected COVID-19. Questions were initially developed in English and then translated into Bahasa Indonesia (Supplementary material S3) by native speakers. Later, another translator conducted back-translation to ensure accuracy.

### 2.4 Validity and reliability

To ensure the validity and reliability of the questionnaire, five experts in public health, epidemiology, pulmonology, infection, and clinical medicine assessed the content validity. The internal consistency of all indexes was calculated using test–retest reliability. The parameters in this questionnaire had excellent validity and reliability, with a content validity index of 0.951 and a test–retest reliability coefficient of 0.900.

### 2.5 Ethical considerations

The Health Research Ethics Commission of the Faculty of Public Health of the University of Muhammadiyah Jakarta approved the study protocol (10.340.B/KEPK-FKMUMJ/IX/2021). The questionnaire was accompanied by a letter that explained the purpose of the study and provided informed consent. Participants were assured that their responses would be kept confidential. Returning the completed questionnaire was considered as giving consent to participate in the study. Participants were also given the option to return the questionnaire anonymously.

### 2.6 Statistical analyses

The Likert-type scale was dichotomized for further analyses to enhance the interpretability of our results, which can be done on relatively similar characteristics of respondents (26). For the assessment of knowledge and attitudes, a rating of strongly agree or agree was regarded as a favorable response and that of neutral, disagree, or strongly disagree as an unfavorable response. For the assessment of practices and institutional support, a rating of often or always was regarded as favorable and that of never, rarely, or sometimes as unfavorable. Sufficient knowledge, positive attitudes, and healthy practices were indicated if the number of favorable answers given by the respondent is greater than or equal to the median number of all questions. Psychological distress was categorized using Kessler’s scale, which differentiates distress into three categories: moderate to severe distress (score: 25–50), mild distress (score: 20–24), and no distress (score: 10–19) (27).

All statistical analyses were conducted using STATA version 14.1 for Windows (StataCorp LP, College Station, TX, United States). Categorical variables were analyzed using the chi-square test, likelihood ratio, or Fisher’s exact test when appropriate. A multiple logistic regression model was used to determine the differences in perceptions toward COVID-19 by adjusting the demographic factors, including sex, age, profession, education, faculty position, and managerial position. Significant differences were defined as *p* < 0.05.

## 3 Results

### 3.1 Demographic data of participants

We included 402 and 584 valid responses from the first and second surveys, respectively, in the analysis. The demographic characteristics of the enrolled participants are summarized in Table 1. Overall, most participants were women, aged ≥30 years, were nurses, had diploma-level education, had no faculty or managerial position, and lived with children or older adults. There was a slight difference in the profession between HCP recruited in the Omicron and post-Omicron eras. The other demographic characteristics did not carry significant difference, including sex, age, education, faculty position, director position, and living with children or older adults.

**Table 1:** Demographic characteristics of the enrolled participants.

Characteristics	Omicron era		post-Omicron era		*p* value
	*N* = 402		*N* = 584		
Sex					
Men	83	20.6%	117	20.0%	0.814
Women	319	79.4%	467	80.0%	
Age (years old)					
20–29	131	32.6%	224	38.4%	0.197
30–39	160	39.8%	228	39.0%	
40–49	90	22.4%	108	18.5%	
>50	21	5.2%	24	4.1%	
Profession					
Physician	25	6.2%	25	4.3%	0.030*
Nurse	262	65.2%	348	59.6%	
Paramedic	115	28.6%	211	36.1%	
Education					
Senior/Vocational High School	10	2.5%	23	3.9%	0.620
Diploma	254	63.2%	345	59.1%	
Bachelor’s degree	60	14.9%	94	16.1%	
Professional Education Program	68	16.9%	107	18.3%	
Master/Specialist Education Program	10	2.5%	15	2.6%	
Faculty position					
Yes	15	3.7%	18	3.1%	0.578
No	387	96.3%	566	96.9%	
Director position					
Yes	33	8.2%	61	10.5%	0.240
No	369	91.8%	523	89.5%	
Living with children or older adults					
Yes	264	65.7%	371	63.5%	0.490
No	138	34.3%	213	36.5%	

* significant difference.

The COVID-19-related characteristics of the enrolled participants are presented in Table 2. The proportion of HCP receiving ≥3 doses of COVID-19 vaccine increased from 64.7 to 86.1% from the Omicron to post-Omicron era, respectively. Most HCP participants had treated patients with confirmed or suspected COVID-19. In addition, HCP in the Omicron era more often contacted or handled specimens of patients with confirmed or suspected COVID-19 than those in the post-Omicron era.

**Table 2:** COVID-19-related characteristics of the enrolled participants.

Characteristics	Omicron era		post-Omicron era		*p* value
COVID-19 vaccination					
Yes – ≥3 doses	260	64.7%	503	86.1%	<0.001*
Yes – 2 doses	134	33.3%	72	12.3%	
Yes – 1 dose	8	2.0%	8	1.4%	
No	0	0.0%	1	0.2%	
Ever treated patients with confirmed or suspected COVID-19					
Yes	294	73.1%	400	68.5%	0.117
No	108	26.9%	184	31.5%	
Ever contacted or handled specimens of patients with confirmed or suspected COVID-19					
Yes	242	60.2%	298	51.0%	0.004*
No	160	39.8%	286	49.0%	

*significant difference.

### 3.2 Knowledge, attitudes, and practices regarding COVID-19

Table 3 presents the KAP results of the Omicron and post-Omicron eras for comparison. Favorable answers in 8 questions for knowledge, 7 questions for attitudes, and 10 questions for practices were defined to be indicative of sufficient knowledge, a positive attitude, and sound practices, respectively. Positive attitudes were significantly more common among HCP in the Omicron era than in the post-Omicron era. No significant differences were observed in sufficient knowledge and healthy practices between the two surveys.

**Table 3:** KAP of HCP in Indonesia.

KAP	Omicron era		post-Omicron era		*p* value
Sufficient knowledge	252	62.7%	357	61.1%	0.621
Positive attitudes	283	70.4%	349	59.8%	0.001*
Healthy practices	259	64.4%	377	64.5%	0.967

*significant difference.

### 3.3 Institutional support for COVID-19

Table 4 presents the levels of institutional support for COVID-19 during Omicron and post-Omicron eras. During the Omicron era, 90.0% of HCP reported having enough surgical masks, indicating a high level of availability. In the post-Omicron era, this percentage slightly decreased to 87.8%. Regarding N95 masks, 66.2% of HCP reported having a sufficient number during the Omicron era, whereas the percentage decreased to 61.6% in the post-Omicron era. Similar patterns were observed for sterile rubber gloves (from 55.2% to 50.9%) and disposable isolation clothing (from 53.5 %to 47.8%). Regarding the redesigning of crowded meeting rooms and environments, 68.9% of HCP reported such measures during the Omicron era, and the percentage slightly decreased to 66.8% in the post-Omicron era. None of these differences were statistically significant. The only variable that significantly decreased was the availability of face shields and protective eyewear from 62.7% in the Omicron era to 55.6% in the post-Omicron era.

**Table 4:** Institutional supports for COVID-19.

Institutional support	Omicron era		post-Omicron era		*p* value
	*N* = 402		*N* = 584		
I have enough surgical masks	364	90.0%	513	87.8%	0.281
I have enough N95 masks	266	66.2%	360	61.6%	0.147
I have enough face shields and protective eyewear	252	62.7%	325	55.6%	0.028*
I have enough sterile rubber gloves	222	55.2%	297	50.9%	0.177
I have enough disposable isolation clothing	215	53.5%	279	47.8%	0.078
My workplace is redesigning crowded meeting rooms and environments	277	68.9%	390	66.8%	0.483

*significant difference.

### 3.4 Psychological distress during COVID-19

Table 5 displays the prevalence of psychological disorders categorized by Kessler’s scale. During the Omicron era, 64.9% of HCP had no psychological distress, which decreased to 56.5% in the post-Omicron era. Consistently, the proportion of HCP experiencing mild psychological distress increased from 8.2% to 11.5%, respectively, and those experiencing moderate to severe psychological distress also increased from 26.9% to 32.0%, respectively. Overall, the HCP experienced significantly increased psychological distress in the post-Omicron era than in the Omicron era.

**Table 5:** Psychological distress during the COVID-19 pandemic assessed using the Kessler’s scale.

Psychological disorder	Omicron era		post-Omicron era		*p* value
	*N* = 402		*N* = 584		
Moderate to severe	108	26.9%	187	32.0%	0.024*
Mild	33	8.2%	67	11.5%	
None	261	64.9%	330	56.5%	

*p < 0.05.

### 3.5 Impact of COVID-19 pandemic among HCP

Table 6 presents the impacts of the COVID-19 pandemic on the lifestyle of HCP. Overall, the most common impact of COVID-19 pandemic was work, followed by diet behavior, family life, income, transportation, and leisure. Nearly two thirds of HCP (65.4% in the Omicron era and 61.1% in the post-Omicron era) always or often experienced work-related impacts. For transportation, a half of HCP always or often experienced transportation-related impacts during the Omicron and post-Omicron eras. For leisure activities, family life, diet behavior, and income, the percentages of HCP reporting never or rarely, sometimes, and always or often having experiences of the impact of the pandemic remained relatively stable during and after the Omicron pandemic. These differences were not statistically significant.

**Table 6:** Impact of COVID-19 pandemic on the lifestyle of HCP.

Impact on lifestyle	Omicron era		post-Omicron era		*p* value
	*N* = 402		*N* = 584		
Transportation, commute					
Never/Rarely	120	29.8%	163	27.9%	0.624
Sometimes	80	20.0%	130	22.3%	
Always/Often	202	50.2%	291	49.8%	
Work					
Never/Rarely	65	16.2%	95	16.3%	0.259
Sometimes	74	18.4%	132	22.6%	
Always/Often	263	65.4%	357	61.1%	
Leisure					
Never/Rarely	105	26.1%	154	26.3%	0.948
Sometimes	136	33.8%	202	34.3%	
Always/Often	161	40.1%	228	39.4%	
Family life					
Never/Rarely	100	24.9%	153	26.2%	0.666
Sometimes	75	18.7%	118	20.2%	
Always/Often	227	56.5%	313	53.6%	
Diet behavior					
Never/Rarely	67	16.7%	101	17.3%	0.505
Sometimes	81	20.1%	134	22.9%	
Always/Often	254	63.2%	349	59.8%	
Income					
Never/Rarely	82	20.4%	122	20.9%	0.422
Sometimes	104	25.9%	171	29.3%	
Always/Often	216	53.7%	291	49.8%	

### 3.6 Multiple logistic regression analyses

Table 7 compares the results of KAP and psychological distress between the Omicron and post-Omicron eras after adjustment for demographic characteristics. Positive attitudes were less prevalent among HCP in the post-Omicron era than in the Omicron era. In addition, psychological distress was more common among HCP in the post-Omicron era than in the Omicron era.

**Table 7:** Multiple logistic regression analyses for KAP and psychological distress.

KAP	Round	OR (95% CI)	*p* value
Sufficient knowledge	Omicron era	Ref	
	post-Omicron era	0.976 (0.747–1.275)	0.860
Positive attitudes	Omicron era	Ref	
	post-Omicron era	0.626 (0.476–0.823)	0.001**
Healthy practices	Omicron era	Ref	
	post-Omicron era	1.080 (0.822–1.418)	0.580
Psychological distress	Omicron era	Ref	
	post-Omicron era	1.371 (1.044–1.799)	0.023*

*p < 0.05, **p < 0.01.

## 4 Discussion

This study assessed the changes in perceptions toward COVID-19 from before the Omicron outbreak to the post-Omicron era among HCP in Indonesia. Our results revealed a significant decline in positive attitudes and increase in psychological distress between HCP in the Omicron and post-Omicron eras. To our knowledge, this is the first study to investigate the changes in perceptions toward the impact of COVID-19 between the Omicron and post-Omicron eras in Indonesia.

Our results demonstrate a notable increase in psychological distress after the Omicron outbreak. The findings are similar to those of Syamlan et al. (13) reporting a higher prevalence of depression, anxiety, and stress among HCP in Indonesia in 2022 than that observed in studies conducted in 2020–2021. They attributed the cause of the discrepancy to the time difference in data collection. Our study further extended their inquiry by using the same questionnaire to investigate the same group of HCP from the end of 2021 (Omicron era) to the end of 2022 (post-Omicron era). Although the disease burden significantly declined and the government’s COVID-19 control measures loosened after the Omicron outbreak, the psychological stress of the HCP increased. This may be because HCP in Indonesia were under considerable pressure during the Omicron wave, leading to a fear of another outbreak. This also explains our finding of a decline in the prevalence of positive attitudes. Our results suggest a need for targeted interventions to maintain positive attitudes toward COVID-19 among HCP, especially as the pandemic probably continues to evolve. Efforts to improve attitudes to prevent COVID-19 transmission can be made through online training or information dissemination, which can avoid adding to the workload of HCP (28).

Our study revealed no significant differences in knowledge and practices between the first and second surveys, implying consistency in the knowledge and behaviors of HCP toward COVID-19. This might be due to the abundance of information available on the topic and the prolonged duration of the pandemic, which led to the adoption of new habits by HCP (29–32). However, positive attitudes toward COVID-19 significantly declined among HCP after the Omicron outbreak, which may be associated with the rising psychological distress. Similar with our result, a prospective study in Singapore focusing on perceived stress and job burnout among HCP indicated a mild increase over a six-month period, persisting even after the conclusion of lockdown measures (33).

Institutional support refers to the supportive measures provided by hospitals to meet the physical, emotional, and psychological needs of HCP (34, 35). Our findings indicate that although some aspects of institutional support for COVID-19 slightly declined in the post-Omicron era, such as the availability of face shields and protective eyewear, overall, the differences were not statistically significant for most of the evaluated factors. However, continually assessing and improving institutional support is essential to ensure the safety and well-being of individuals within these environments, especially given that institutional support mediates the relationship between COVID-19 stressors and psychological distress (36). Meanwhile, a global systematic review revealed that the adverse working conditions stemming from emergency responses during the pandemic have emerged as significant contributors to HCP’s turnover intention (37).

In our cohort of HCP, the impact of the COVID-19 pandemic on transportation, work, leisure, family life, diet behavior, and income did not significantly change between the Omicron and post-Omicron eras. A longitudinal over two years during the COVID-19 pandemic showed that sleep quality among HCP worsened (38). Notably, the day-to-day realities for HCP did not exhibit substantial improvements. HCP continued to face challenges throughout the study period and may have required continued support to navigate the effects of the pandemic on various aspects of their lives. Our findings underscore the impact of the COVID-19 did not fade right away after the Omicron pandemic. Future pandemic preparedness plans should prioritize the establishment of sustained support systems for HCP.

This study has some limitations. First, this use of self-reported data may be subject to bias or social desirability effects. We sought participants through their managers of heads of unit to minimize potential bias from the voluntary nature of online surveys. Second, the HCP were recruited from four hospitals in Indonesia, which may limit the generalizability of the findings to other healthcare settings or regions. Nevertheless, the four hospitals we selected were located in three different islands of Indonesia, which may reduce the geographical bias. Third, the respondents did not overlap completely across the two questionnaire surveys because we did not link the data to individual HCP participants. Nevertheless, the influence of this should be minimal because of comparable demographic characteristics between the two groups and the use of a multiple logistic regression model to reduce the potential bias from the demographic characteristics. Fourth, we focused on perceptions and practices related to COVID-19 only among HCP and did not examine the impact of these perceptions and practices on patient outcomes or healthcare system capacity. We also did not assess the impact of external factors, such as government policies or media coverage, on the COVID-19-related perceptions and practices of HCP.

## 5 Conclusion

The current study demonstrated a significant increase in psychological distress and decrease in positive attitudes toward COVID-19 after the Omicron outbreak among HCP in Indonesia, necessitating further interventions and support systems to mitigate the psychological effects of the pandemic and promote overall well-being. HCP should be provided with education and training to ensure that they have up-to-date knowledge related to the evolving dynamics of COVID-19 mutations. This preparation is also crucial to enhance vigilance and equip them effectively for potential future outbreaks, whether on an epidemic or pandemic scale. Furthermore, measures should be taken to ensure an adequate and consistent supply of essential protective equipment, offer comprehensive mental health support services, address work-related impacts through workload management, ensure appropriate staffing, and support work environments to reduce stress and improve the well-being of HCP.

## Data availability statement

The raw data supporting the conclusions of this article will be made available by the authors, without undue reservation.

## Ethics statement

The studies involving humans were approved by The Health Research Ethics Commission of the Faculty of Public Health of the University of Muhammadiyah Jakarta (10.340.B/KEPK-FKMUMJ/IX/2021). The studies were conducted in accordance with the local legislation and institutional requirements. The participants provided their written informed consent to participate in this study.

## Author contributions

MM: Conceptualization, Data curation, Formal analysis, Methodology, Writing – original draft, Writing – review & editing. YHW: Conceptualization, Data curation, Formal analysis, Methodology, Writing – original draft, Writing – review & editing. YWC: Conceptualization, Data curation, Methodology, Writing – original draft, Writing – review & editing. HYC: Conceptualization, Data curation, Methodology, Writing – review & editing.
