# Supplementary material for: Perceptions of COVID-19 during and after the Omicron outbreak among healthcare personnel in Indonesia
Source: Front Public Health. 2024 Jan 8;11:1321045. doi: 10.3389/fpubh.2023.1321045 (PMC10800601; doi:10.3389/fpubh.2023.1321045)
Supplement: Supplementary file 1 [file Table_1.DOCX]

Supplementary Material

Perceptions of COVID-19 During and After the Omicron Outbreak among Healthcare Personnel in Indonesia

Mohammad Ainul Maruf, Yi-Hao Weng, Ya-Wen Chiu^*^, Hung-Yi Chiou^*^

*** Correspondence:** Corresponding Author: [bettychiu@gap.kmu.edu.tw](mailto:bettychiu@gap.kmu.edu.tw) and [hychiou@tmu.edu.tw](mailto:hychiou@tmu.edu.tw)

# S1 Government policy toward the COVID-19 pandemic in Indonesia during the two study periods (December 2021 to February 2022 and August to October 2022).

| **Government policy** | **Omicron era** | **post-Omicron era** |
| --- | --- | --- |
| Quarantine | Imposed | Lifted |
| Dining at restaurant | Partially restricted | Not restricted |
| Transportation | Partially restricted | Not restricted |
| People-gathering | Partially restricted | Not restricted |
| Religious activity | Partially restricted | Not restricted |
| Meeting | Partially restricted | Not restricted |
| Public wedding/funeral | Partially restricted | Not restricted |
| Wearing mask in public | Mandatory | Encouraged |
| Work | Work from home is encouraged | Work from office |

# S2 Questionnaires in English

**A-Demographics**

1. Sex:

- Male
- Female
- Not applicable or not willing to answer

1. Age: ________________________________________________ years old
2. Your highest education level:

□ Senior/Vocational High School
□ Diploma
□ Bachelor Degree
□ Professional Education Program

□ Master Degree or Specialist Education Program
□ Doctoral Degree or Subspecialty Education Program

1. Your profession and work environment:

- Medical doctor/Physician
- Subspecialist doctor/Consultant
- Specialist doctor
- Resident doctor
- General practitioner
- Intern
- Co-assistant
- Nurse
- Specialist nurse
- Professional nurse
- Vocational nurse
- Other type of health worker: ________________ (please provide details)

1. How long have you worked at the current hospital/institution? _________ year(s)
2. Do you hold any managerial position?

- □ Yes, as _____________ (please specify)
- □ No

1. Do you hold any teaching position?

- □ Yes
- □ No

1. During the COVID-19 pandemic, did you ever treat patients with confirmed or suspected COVID-19?

- Yes, treated patients with confirmed COVID-19
- Yes, treated patients with suspected COVID-19
- None
- Both

1. During the COVID-19 pandemic, did you ever contact or handle specimens or samples of patients with confirmed or suspected COVID-19?

- Handled specimens/samples of patients with confirmed COVID-19
- Handled specimens/samples of patients with suspected COVID-19
- None
- Both

1. Do you have child/children (<18 years) or older adults (>65 years) living with you?

- Yes, child/children
- Yes, older adults
- None
- Both

1. Have you ever had the COVID-19 vaccine?

□ Yes

How many injections did you receive?

□ 1

□ 2

□ 3

□ No

**B-Content**

I. Regarding COVID-19, do you agree with the following statements? (self-assessment)

|  | **Strongly Agree** | **Agree** | **Neutral/No Comment** | **Disagree** | **Strongly Disagree** |
| --- | --- | --- | --- | --- | --- |
| 1-1. The main clinical symptoms of COVID-19 include fever, fatigue, dry cough, and pain. | □ | □ | □ | □ | □ |
| 1-2. Nasal congestion, rhinorrhea, and sneezing are rare in patients with COVID-19. | □ | □ | □ | □ | □ |
| 1-3. Dysgeusia and anosmia are among the symptoms of COVID-19. | □ | □ | □ | □ | □ |
| 1-4. So far, no definite and effective treatment exists for COVID-19. Early/immediate supportive care can help some patients recover. | □ | □ | □ | □ | □ |
| 1-5. Patients with advanced age, chronic disease, respiratory disease, or obesity have a higher risk of COVID-19. | □ | □ | □ | □ | □ |
| 1-6. Consuming or having contact with wildlife can increase the risk of COVID-19. | □ | □ | □ | □ | □ |
| 1-7. COVID-19 can be transmitted through droplets or aerosols from the respiratory tract of infected patients. | □ | □ | □ | □ | □ |
| 1-8. Quarantine or isolation is effective in reducing the spread of COVID-19. | □ | □ | □ | □ | □ |

II. Regarding COVID-19, please rate the following statements according to your own perception. (Self-assessment; not about actions in individual countries)

|  | **Strongly Agree** | **Agree** | **Neutral/No Comment** | **Disagree** | **Strongly Disagree** |
| --- | --- | --- | --- | --- | --- |
| 2-1. The public in general can use surgical masks to prevent the transmission of COVID-19. | □ | □ | □ | □ | □ |
| 2-2. Children and youth do not need to take precautions against COVID-19. *(reversed)* | □ | □ | □ | □ | □ |
| 2-3. During the COVID-19 pandemic, people should avoid using public transportation and social gatherings. | □ | □ | □ | □ | □ |
| 2-4. People who have a history of contact with a patient with confirmed COVID-19 should immediately be quarantined for 14 days. | □ | □ | □ | □ | □ |
| 2-5. I understand the precautions against COVID-19 in my country. | □ | □ | □ | □ | □ |
| 2-6. I am willing to comply with the precautions against COVID-19 in my country. | □ | □ | □ | □ | □ |
| 2-7. I believe in the COVID-19 prevention recommendations by the World Health Organization (WHO) | □ | □ | □ | □ | □ |

III. During the COVID-19 pandemic, have you practiced the following behaviors in your daily life? (Self-assessment)

|  | **Always** | **Often** | **Sometimes** | **Rarely** | **Never** |
| --- | --- | --- | --- | --- | --- |
| 3-1. Avoid social contact (e.g., eating out with friends) | □ | □ | □ | □ | □ |
| 3-2. Avoid social gatherings (less than or equal to 100 people) | □ | □ | □ | □ | □ |
| 3-3. Avoid mass gatherings (more than 100 people) | □ | □ | □ | □ | □ |
| 3-4. Avoid business meetings or turn them into online meetings | □ | □ | □ | □ | □ |
| 3-5. Avoid crowded place | □ | □ | □ | □ | □ |
| 3-6. Wear a mask in public | □ | □ | □ | □ | □ |
| 3-7. Implement social distancing in public places | □ | □ | □ | □ | □ |
| 3-8. Avoid eating out | □ | □ | □ | □ | □ |
| 3-9. Use gloves | □ | □ | □ | □ | □ |
| 3-10. Wash your hands regularly and use hand sanitizer when needed | □ | □ | □ | □ | □ |

IV. Kessler Psychological Distress Scale (K10)

|  | **All of the time** | **Most of the time** | **Some of the time** | **A little of the time** | **None of the time** |
| --- | --- | --- | --- | --- | --- |
| 4-1. In the past 4 weeks, how often have you felt tired for no apparent reason? | □ | □ | □ | □ | □ |
| 4-2. In the past 4 weeks, how often have you felt nervous or anxious? | □ | □ | □ | □ | □ |
| 4-3. In the past 4 weeks, how often have you felt so nervous or anxious that nothing could calm you down? | □ | □ | □ | □ | □ |
| 4-4. In the past 4 weeks, how often have you felt a sense of hopelessness? | □ | □ | □ | □ | □ |
| 4-5. In the past 4 weeks, how often have you felt restless? | □ | □ | □ | □ | □ |
| 4-6. In the past 4 weeks, how often have you felt so restless that you couldn't sit still? | □ | □ | □ | □ | □ |
| 4-7. In the past 4 weeks, how often have you felt depressed? | □ | □ | □ | □ | □ |
| 4-8. In the past 4 weeks, how often have you felt that things required effort? | □ | □ | □ | □ | □ |
| 4-9. In the past 4 weeks, how often have you felt so sad that nothing could comfort you? | □ | □ | □ | □ | □ |
| 4-10. In the past 4 weeks, how often have you felt worthless? | □ | □ | □ | □ | □ |

**V.** During the COVID-19 pandemic, how would you rate the extent of changes made to any of the following aspects? (Self-assessment)

|  | **All of the time** | **Most of the time** | **Some of the time** | | **A little of the time** | | **None of the time** | |
| --- | --- | --- | --- | --- | --- | --- | --- | --- |
| 5-1. Transportation and routine travel | □ | □ | □ | □ | | □ | |  |
| 5-2. Work (including shift work and other departmental support) | □ | □ | □ | □ | | □ | |  |
| 5-3. Leisure time | □ | □ | □ | □ | | □ | |  |
| 5-4. Family life (including taking care of children) | □ | □ | □ | □ | | □ | |  |
| 5-5. Dietary habit | □ | □ | □ | □ | | □ | |  |
| 5-6. Income | □ | □ | □ | □ | | □ | |  |

# S3 Questionnaires in Bahasa Indonesia

**A-Demografi**

1. Jenis Kelamin:

- Laki-laki
- Perempuan
- Tidak berlaku atau tidak bersedia menjawab

1. Usia: ________________________________________________ tahun
2. Jenjang pendidikan tertinggi Anda:

□ Sekolah Menengah Kejuruan
□ Diploma
□ Sarjana (S1)
□ Program Pendidikan Profesi

□ Magister (S2) atau Pendidikan Spesialis
□ Doktor (S3) atau Pendidikan Subspesialis

1. Profesi dan lingkungan kerja Anda:

- Dokter
- Dokter subspesialis/konsultan
- Dokter spesialis
- Dokter residen
- Dokter umum
- Dokter internship
- Dokter koas
- Ners (Perawat)
- Ners spesialis
- Ners profesi
- Ners vokasi atau ahli madya
- Tenaga kesehatan lainnya: ________________ (mohon diisi)

1. Berapa lama Anda bekerja di rumah sakit/institusi sekarang? _________ tahun
2. Apakah Anda memegang posisi manajerial?

- □ Ya, sebagai _____________ (mohon diisi)
- □ Tidak

1. Apakah Anda memegang posisi mengajar?

- □ Ya
- □ Tidak

1. Selama masa pandemi COVID-19, apakah Anda pernah merawat pasien terkonfirmasi atau suspek COVID-19?

- Ya, merawat pasien terkonfirmasi COVID-19
- Ya, merawat pasien terduga/suspek COVID-19
- Keduanya tidak
- Keduanya ya

1. Selama masa pandemi COVID-19, apakah Anda pernah kontak atau menangani spesimen atau sampel pasien terkonfirmasi atau suspek COVID-19?

- Menangani sampel pasien terkonfimasi COVID-19
- Menangani sampel pasien terduga/suspek COVID-19
- Tidak keduanya
- Ya keduanya

1. Apakah Anda memiliki anak (<18 tahun) atau lansia (>65 tahun) yang tinggal bersama Anda?

- Ya, mempunyai anak
- Ya, mempunyai lansia
- Tidak keduanya
- Ya keduanya

1. Apakah Anda pernah mendapatkan vaksin COVID-19?

□ Ya

Berapa banyak suntikan yang Anda terima?

□ 1 suntikan

□ 2 suntikan

□ 3 suntikan

□ Tidak

**B-Konten**

I. Mengenai COVID-19, apakah Anda setuju dengan pernyataan berikut? (penilaian diri)

|  | **Sangat setuju** | **Setuju** | **Netral/ Tidak ada komentar** | **Tidak setuju** | **Sangat tidak setuju** |
| --- | --- | --- | --- | --- | --- |
| 1-1. Gejala klinis utama COVID-19 termasuk demam, kelelahan, batuk kering, dan nyeri. | □ | □ | □ | □ | □ |
| 1-2. Hidung tersumbat, rinorea, dan bersin jarang terjadi diantara pasien COVID-19. | □ | □ | □ | □ | □ |
| 1-3. Disgeusia dan anosmia adalah salah satu gejala klinis COVID-19. | □ | □ | □ | □ | □ |
| 1-4. Hingga saat ini, belum ada pengobatan yang pasti dan efektif untuk COVID-19. Perawatan suportif dini/segera dapat membantu sebagian pasien pulih. | □ | □ | □ | □ | □ |
| 1-5. Pasien dengan usia tua, penyakit kronis, penyakit pernapasan, atau obesitas memilki risiko lebih tinggi untuk menderita akibat infeksi COVID-19. | □ | □ | □ | □ | □ |
| 1-6. Makan atau bersentuhan dengan satwa liar dapat meningkatkan risiko terinfeksi COVID-19. | □ | □ | □ | □ | □ |
| 1-7. COVID-19 dapat menginfeksi orang melalui droplet atau aerosol dari saluran pernapasan pasien COVID-19. | □ | □ | □ | □ | □ |
| 1-8. Karantina atau isolasi merupakan cara efektif mengurangi penyebaran COVID-19 kepada orang lain. | □ | □ | □ | □ | □ |

II. Mengenai COVID-19, silakan beri nilai pada pernyataan berikut menurut persepsi Anda sendiri. (Penilaian diri; bukan mengenai tindakan di masing-masing negara)

|  | **Sangat setuju** | **Setuju** | **Netral/ Tidak ada komentar** | **Tidak setuju** | **Sangat tidak setuju** |
| --- | --- | --- | --- | --- | --- |
| 2-1. Masyarakat umum dapat memakai masker bedah untuk mencegah infeksi COVID-19. | □ | □ | □ | □ | □ |
| 2-2. Anak-anak dan orang muda tidak perlu melakukan tindakan pencegahan terhadap COVID-19. *(berkebalikan)* | □ | □ | □ | □ | □ |
| 2-3. Selama pandemi COVID-19, masyarakat harus menghindari penggunaan transportasi umum dan menghadiri perkumpulan sosial. | □ | □ | □ | □ | □ |
| 2-4. Orang-orang yang memiliki riwayat kontak dengan pasien terkonfirmasi COVID-19 harus segera dikarantina selama 14 hari. | □ | □ | □ | □ | □ |
| 2-5. Saya memahami tindakan pencegahan terhadap COVID-19 di negara saya. | □ | □ | □ | □ | □ |
| 2-6. Saya bersedia untuk mematuhi tindakan pencegahan terhadap COVID-19 di negara saya. | □ | □ | □ | □ | □ |
| 2-7. Saya bersedia untuk meyakini rekomendasi pencegahan COVID-19 yang diumumkan oleh WHO (Badan Kesehatan Dunia) | □ | □ | □ | □ | □ |

III. Selama pandemi COVID-19, apakah Anda melakukan perilaku berikut ini dalam kehidupan Anda sehari-hari? (Penilaian diri)

|  | **Selalu** | **Sering** | **Kadang-kadang** | **Jarang** | **Tidak pernah** |
| --- | --- | --- | --- | --- | --- |
| 3-1. Menghindari kontak sosial (misalnya, makan bersama bersama teman) | □ | □ | □ | □ | □ |
| 3-2. Menghindari perkumpulan sosial (kurang atau sama dengan 100 orang) | □ | □ | □ | □ | □ |
| 3-3. Menghindari perkumpulan massa (lebih dari 100 orang) | □ | □ | □ | □ | □ |
| 3-4. Menghindari pertemuan bisnis atau mengubahnya menjadi pertemuan online | □ | □ | □ | □ | □ |
| 3-5. Menghindari tempat-tempat keramaian | □ | □ | □ | □ | □ |
| 3-6. Memakai masker di tempat-tempat umum | □ | □ | □ | □ | □ |
| 3-7. Menerapkan jaga jarak sosial di tempat-tempat umum | □ | □ | □ | □ | □ |
| 3-8. Menhindari makan di luar rumah | □ | □ | □ | □ | □ |
| 3-9. Menggunakan sarung tangan | □ | □ | □ | □ | □ |
| 3-10. Mencuci tangan secara rutin dan menggunakan hand sanitizer ketika dibutuhkan. | □ | □ | □ | □ | □ |

IV. . Skala Distres Psikologis Kessler (K10)

|  | **Setiap saat** | **Sering kali** | **Beberapa kali** | **Sesekali** | **Tidak pernah** |
| --- | --- | --- | --- | --- | --- |
| 4-1. Dalam 4 minggu terakhir, seberapa sering Anda merasa lelah tanpa alasan yang jelas? | □ | □ | □ | □ | □ |
| 4-2. Dalam 4 minggu terakhir, seberapa sering Anda merasa gugup atau cemas? | □ | □ | □ | □ | □ |
| 4-3. Dalam 4 minggu terakhir, seberapa sering Anda merasa sangat gugup atau cemas sehingga tidak ada yang bisa menenangkan Anda? | □ | □ | □ | □ | □ |
| 4-4. Dalam 4 minggu terakhir, seberapa sering Anda merasa putus asa? | □ | □ | □ | □ | □ |
| 4-5. Dalam 4 minggu terakhir, seberapa sering Anda merasa gelisah atau resah? | □ | □ | □ | □ | □ |
| 4-6. Dalam 4 minggu terakhir, seberapa sering Anda merasa sangat gelisah sehingga tidak bisa duduk terdiam? | □ | □ | □ | □ | □ |
| 4-7. Dalam 4 minggu terakhir, kira-kira seberapa sering Anda merasa tertekan/depresi? | □ | □ | □ | □ | □ |
| 4-8. Dalam 4 minggu terakhir, kira-kira seberapa sering Anda merasa bahwa segala sesuatu memerlukan usaha? | □ | □ | □ | □ | □ |
| 4-9. Dalam 4 minggu terakhir, seberapa sering Anda merasa sangat sedih sehingga tidak ada yang bisa menghibur Anda? | □ | □ | □ | □ | □ |
| 4-10. Dalam 4 minggu terakhir, seberapa sering Anda merasa tidak berharga? | □ | □ | □ | □ | □ |

V. Selama masa pandemi COVID-19, bagaimana Anda menilai perubahan Anda pada hal-hal berikut? (Penilaian diri)

|  | **Setiap saat** | **Sering kali** | **Beberapa kali** | **Sesekali** | **Tidak pernah** |
| --- | --- | --- | --- | --- | --- |
| 5-1. Transportasi, perjalanan rutin | □ | □ | □ | □ | □ |
| 5-2. Pekerjaan (termasuk shift kerja, dukungan departemen lain, dsb.) | □ | □ | □ | □ | □ |
| 5-3. Waktu luang | □ | □ | □ | □ | □ |
| 5-4. Kehidupan keluarga (termasuk mengurus anak) | □ | □ | □ | □ | □ |
| 5-5. Pola makan | □ | □ | □ | □ | □ |
| 5-6. Pemasukan | □ | □ | □ | □ | □ |
